# Increasing the speed of medical image processing in MatLab^®^

**DOI:** 10.2349/biij.3.1.e9

**Published:** 2007-01-01

**Authors:** M Bister, CS Yap, KH Ng, CH Tok

**Affiliations:** 1 School of Electrical and Electronic Engineering, The University of Nottingham, Malaysia Campus, Semenyih, Selangor, Malaysia; 2 Department of Biomedical Imaging, Faculty of Medicine, University of Malaya, Kuala Lumpur, Malaysia

**Keywords:** MatLab^®^, image processing, optimisation, vectorisation, specialisation

## Abstract

MatLab^®^ has often been considered an excellent environment for fast algorithm development but is generally perceived as slow and hence not fit for routine medical image processing, where large data sets are now available e.g., high-resolution CT image sets with typically hundreds of 512x512 slices. Yet, with proper programming practices – vectorization, pre-allocation and specialization – applications in MatLab^®^ can run as fast as in C language. In this article, this point is illustrated with fast implementations of bilinear interpolation, watershed segmentation and volume rendering.

## INTRODUCTION

In recent years, MatLab^®^, the product of MathWorks, has become a popular tool for fast development. Its many Toolboxes, powerful interface and user-friendliness make it a tool of choice in many disciplines, including medical image processing. However, two drawbacks are frequently noted: its low processing speed and wasteful use of memory. These have led many developers and researchers to do a fast development of their application in MatLab^®^ first and then to re-program it in another language for production or distribution (typically C/C++ for the procedural part and Visual Basic for the user interface).

MathWorks has provided a compiler to translate m-files (MatLab^®^ programs) in C/C++ and FORTRAN. However, the translated code preserves the flexibility of MatLab^®^ and hence even the compiled code remains as slow and uses as much memory [1]. The main advantage of translation and compilation is the distribution of the developed application to users or colleagues who do not have a (compatible) MatLab^®^ licence.

In medical image processing, the problems of memory usage and low execution speed are compounded with ever-increasing sizes of data sets. Typical High Resolution Computed Tomography (HRCT) image sets now include hundreds of 512x512 slices, making up an (almost) isotropic volume, which is best handled as one volume for reasons of consistency of results over the third axis [2].

However, it has been found that good programming practices can greatly reduce the processing time. These good programming practices when using MatLab^®^ are advocated in the MatLab^®^ user manuals and help files and include vectorisation of loops and pre-allocation of memory [3-6], as well as function specialisation. Often, a balance between processing speed and memory usage has to be found.

In the literature, a number of examples of MatLab^®^ code optimization in different areas have been reported. Kobbelt [7] described a vectorised MatLab^®^ program for evaluation of box-splines. Menon [8] discussed automated language translation and highlighted the importance of vectorisation (performance enhancements by a factor of 250:1 were reported) and pre-allocation (improved performance by a factor of 7:1). Günther [9] described the impact of vectorisation on Nuclear Magnetic Resonance (NMR) data processing but emphasised the memory requirements for vectorisation. He concluded: “With increasing amounts of computer memory, the concept of data processing in the computer memory will become the method of choice”, which is becoming a reality due to Moore’s Law.

Chauhan [10] reported on the development of high-level problem-solving languages and pointed to the importance of procedure vectorisation and strength reduction, with performance enhancement up to a factor of 3.3:1. Procedure strength reduction can be seen as a specialization of certain procedures with a view on optimising some specific applications of a given procedure.

Higham [11] reported on the use of MatLab^®^ for mathematical calculations. Yang [12] reported on software development for improved farming methods. Pointon [13] applied MatLab^®^ in three-dimensional (3D) dual head coincidence imaging. Lee [14] optimised a system for forecasting flooding as a result of typhoons and storms. All in their respective fields of application reported mainly the advantage of code vectorisation without mentioning pre-allocation or function specialization.

In the present article, the issues reported in the literature are taken up and applied on the specific needs of medical image processing. The main focus is on three areas, namely vectorisation, pre-allocation and specialization. The principles of these three methods will be explained and their effect illustrated. Subsequently, their effect will also be demonstrated on three short algorithms that are widely used in medical image processing, namely bilinear interpolation, watershed segmentation and volume rendering. A follow-up article will discuss the problems of wasteful memory usage by MatLab^®^ as well as techniques for debugging vectorised programs.

## METHODS

### Illustrative examples

#### Vectorisation and pre-allocation

To illustrate the concepts of vectorising and pre-allocation, a simple program will be considered to re-scale a 3D HRCT scan image from bone window – with Hounsfield Units (HU) from -1250 to 250 – to values in the range of 0 to 1 for display as type single or double using the MatLab^®^ function imshow. Although there exist some built-in MatLab^®^ operators to perform this function, this simple case will be considered to illustrate the concepts of pre-allocation and vectorisation.

Persons with C/C++ programming background would probably come up with a code similar to the function in Listing 1. The input image inim is rescaled, so that the range of values from min0 to max0 are rescaled to the range of values from min1 to max1. Comment lines and help lines have been omitted to concentrate on the code.

**Listing 1:**
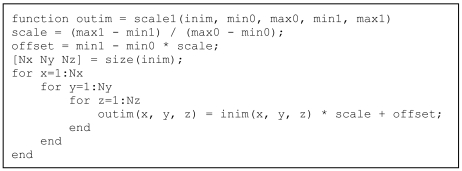
C-like code for rescaling a 3-dimensional array.

As will be discussed in the Results section below, The triple for-loop results in an extremely slow execution. Moreover, the output image outim is built using dynamic memory allocation, a nice and very user-friendly feature of MatLab^®^ but which causes extremely slow execution. A user with background in C will have no problems pre-allocating the output array as in the code in Listing 2 – with a significant increase in processing speed as will be shown in the Results section below, and at the price of a single additional line in the code.

**Listing 2:**
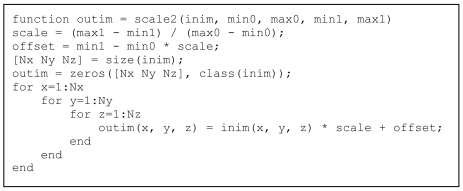
Improved code with pre-allocation for rescaling a 3-dimensional array.

Despite the increase in processing speed, the time-consuming triple for-loop still remains. Vectorising means treating an array as an array and not as a list of elements. In C-like languages, arrays can only be accessed one element at a time while MatLab^®^-like languages allow for manipulating whole arrays at one time. Hence, learning to use MatLab^®^-like languages effectively (with vectorisation) often requires a complete change of thinking pattern. The code in Listing 3 shows the same function as in Listings 1 and 2, but *with* vectorisation. Please note that the code becomes shorter and more readable. As shown in the Results section below, the execution time is also shorter. In this case, no pre-allocation is required as the whole array is processed at one time.

**Listing 3:**
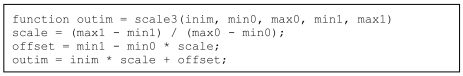
Improved code with vectorisation for rescaling a 3-dimensional array.

#### Vectorisation of conditional statements

Vectorisation of conditional statements is usually done with the find operator in MatLab^®^ whereby the condition is input in the find statement, which returns the indices of the elements on which the conditional operation should be applied. To illustrate, the above scaling operation will be done on pixels with HU within the range of min0 to max0; all the pixels with HU lower than min0 will have an output value of min1 while those with HU higher than max0 will have an output value of max1. Again, there are easier ways to do this in MatLab^®^ but this simple case illustrates the vectorisation of conditional statements. Listings 4 and 5 show the code *without* and *with* vectorisation.

**Listing 4:**
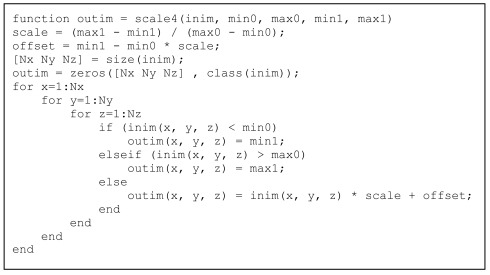
Code with conditional statement for rescaling a 3-dimensional array, *without* vectorisation.

**Listing 5:**
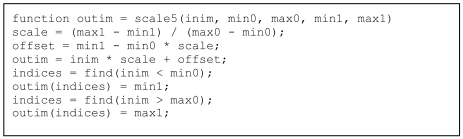
Code with conditional statement for rescaling a 3-dimensional array, *with* vectorisation.

Again, the code *with* vectorisation is much shorter, and as shown in the Results section below, it also runs much faster. The code can be further optimised if the indices on which to apply the conditional operator are not explicitly calculated. This is done in the code in Listing 6. Results are shown in the Results section below and discussed in the following Discussion section. It can be observed that the code is again more compact and yet it retains the same level of readability.

**Listing 6:**
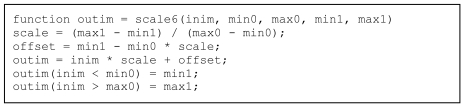
Code with conditional statement for rescaling a 3-dimensional array, with vectorisation and implicit index calculation.

#### Vectorisation of local neighbourhood operations

Another problem in vectorising code for digital image processing is often related to local neighbourhood operations, whereby the output value of a pixel or element depends, not only on the value of this pixel, but also on the value of the neighbouring pixels. In case this is a linear relationship, the new value being a linear combination of the values of the neighbouring pixels, this operation can be easily implemented as a convolution operator. In the case of a non-linear relationship, it is often worthwhile to look at morphological operators implemented in MatLab^®^ (e.g., the bwmorph operator).

In case the desired operator cannot be implemented using the above-mentioned techniques, it is often possible to write out the local neighbourhood along an additional dimension and to apply the operator along that new dimension. To illustrate, a function was implemented to find local maxima in a 3D array. The existing MatLab^®^ operator imregionalmax was implemented in C using MEX programming (MatLab^®^ callable C and Fortran programs are referred to as MEX-files; MEX stands for MatLab^®^ Executables) and does it very efficiently. However, in this article the operator was re-implemented to illustrate the concept of vectorisation. Listing 7 shows the code *without* vectorisation. The six nested for-loops are immediately noticeable (three to scan all the pixels along the three dimensions and another three to scan the neighbourhood of each pixel), with an additional conditional operator resulting in extremely slow execution. The min and max operators on the ranges of the indices i, j and k accommodate the traditional problems that arise at the borders of the image when applying neighbourhood operators.

**Listing 7:**
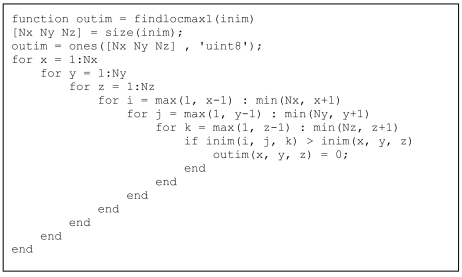
Code for detecting local maxima, *without* vectorisation.

Listing 8 shows the code *with* vectorisation by building a new array with the local neighbourhood written out along a new dimension. An augmented version of the input matrix is formed to circumvent the traditional border problems in neighbourhood operations. The temporary matrix tempim is formed with dimension 3+1 whereby the new dimension lists the values of all the neighbours of the pixels. Of course, this is highly redundant and memory consuming but as shown in the Results section below, the code executes much faster.

**Listing 8:**
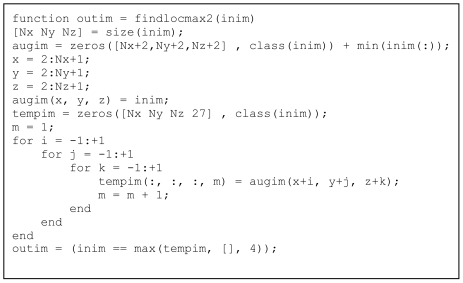
Code for detecting local maxima, with vectorisation by building the local neighbourhood along a new dimension.

A similar approach could be followed to implement operators which are not available in MatLab^®^ e.g., percentile operators whereby the value of the output pixel is the percentile of the values in a neighbourhood of the input array (median, maximum and minimum filters are special cases of percentile filters).

It is obvious that this approach is very wasteful in terms of memory. A compromise could be achieved by vectorising the three outermost loops, replacing the conditional operator with a find statement and keeping the three innermost loops, each of which only runs over three values. The resulting code is shown in Listing 9. The code is a little slower and less versatile (e.g., percentile or median filtering could not be implemented in this way) but the memory requirements are much lower and processing speed is still acceptable, as discussed in the Results section below. Please note that the code is more readable than the one in Listing 8 and closer to the C-like code of Listing 7, making for an easier “translation” from the traditional coding techniques to vectorisation.

**Listing 9:**
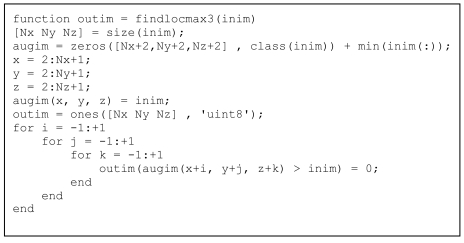
Code for detecting local maxima, with vectorisation while keeping innermost loops and processing using the implicit find command.

#### Specialisation

The specialisation of a function is illustrated in the ‘Medical image processing’ section below with the discussion of a specialised interpolation function for use in medical image processing.

### Medical image processing

#### Bilinear interpolation

Interpolation is an old and well-covered topic in digital image processing. In medical image processing, bilinear interpolation is often used to zoom into a 2D image or for rendering, for display purposes. The formula for bilinear interpolation of a point (x, y) is given by

(1) $$\begin{array}{c} L\left(x,y\right)=\left(\left\lceil x\right\rceil -x\right)\left(\left\lceil y\right\rceil -y\right)L\left(\left\lfloor x\right\rfloor ,\left\lfloor y\right\rfloor \right)+\\ \left(\left\lceil x\right\rceil -x\right)\left(y-\left\lfloor y\right\rfloor \right)L\left(\left\lfloor x\right\rfloor ,\left\lceil y\right\rceil \right)+\\ \left(x-\left\lfloor x\right\rfloor \right)\left(\left\lceil y\right\rceil -y\right)L\left(\left\lceil x\right\rceil ,\left\lfloor y\right\rfloor \right)+\\ \left(x-\left\lfloor x\right\rfloor \right)\left(y-\left\lfloor y\right\rfloor \right)L\left(\left\lceil x\right\rceil ,\left\lceil y\right\rceil \right) \end{array}$$

and the interpolation points are all the values $\left(x,y\right)=\left(\frac{i}{s},\frac{j}{s}\right)$ lying within the area of the image and with *s* = scale of resizing, *i* and *j* integer.

MatLab^®^ has an excellent interpolation function imresize, which allows for a large number of options such as size of the resulting image OR scaling factor, type of interpolation (nearest neighbour, bilinear, bicubic), choice of length of low-pass filter and of the specific filter in the case of size reduction. The procedure calls for another procedure, tformarray, which performs possibly very advanced interpolation functions (even using an irregular sampling grid). The result is that this routine and all its subroutines are over-generalised – they are too flexible for most usual applications in medical image processing and hence take too much time and memory. Listing 10 lists a routine that does two-dimensional (2D) bilinear interpolation in a very simple and efficient way, applying the concepts of pre-allocation and vectorisation as explained in the ‘Illustrative examples’ section above. As shown in the Results section below, the resulting execution speed is much higher than for the standard MatLab^®^ routine, at the expense of reduced flexibility.

**Listing 10:**
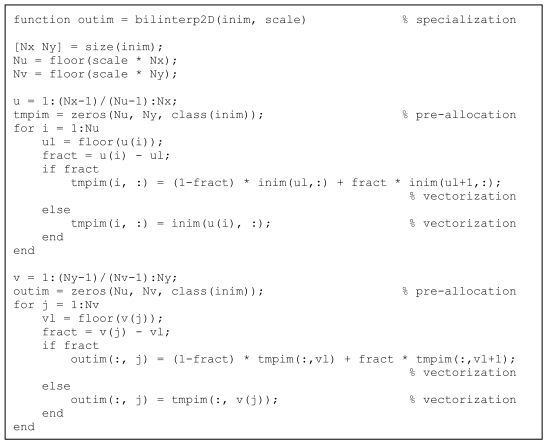
Specialised code for 2D bilinear interpolation.

#### Watershed segmentation

For image segmentation, watershed algorithms [15] have become very popular in all their implementations: applied on gradient image for object delineation, applied on original image for definition of region of interest, applied on distance-transformed images for separation between convex components, associated with filtering and/or merging or in multi-resolution implementation for reduction of over-segmentation, etc. In their steepest-descent approach, each pixel is linked to its neighbour with the lowest intensity and groups of pixels that link together are defined as segments (see the analogy of raindrops falling on a landscape). These algorithms are usually considered time-consuming and are mostly implemented in C. Even the watershed function in MatLab^®^ is implemented as a MEX file. However, by applying the efficient coding techniques described above, it is possible to define a watershed function that is very efficient, as shown in Listing 11. Basic comment lines were left in the code to explain its different steps.

**Listing 11:**
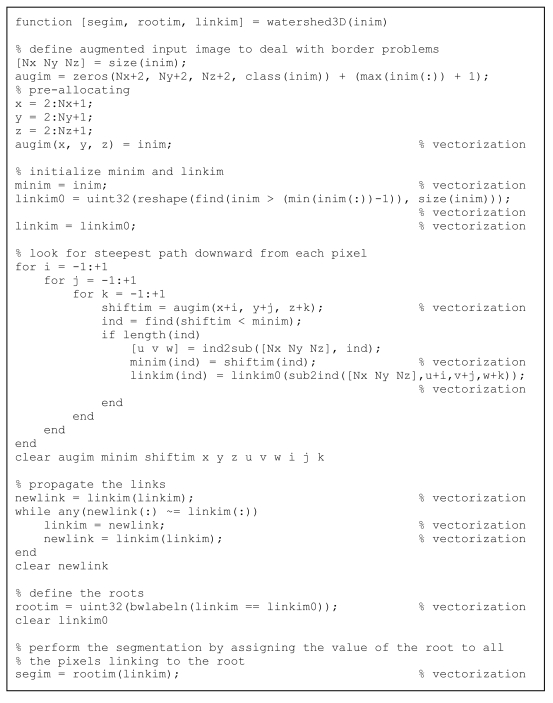
Fast code for watershed segmentation.

As shown in the Results section below, the performance of this routine is competitive with the performance of the native routine in C, which shows that MatLab^®^ programming, when properly done, can be competitive with C programming for speed. Hence, the old adage that MatLab^®^ is only good for fast development but not for speed might have to be overturned.

#### Volume rendering

A third example of efficient coding in MatLab^®^ for medical image processing applications is volume rendering [16-17], as in Listing 12 – again a procedure which is usually considered very time-consuming. Inputs to the function are the original 3D array, the coordinates of the camera (position, tilt and pan, focus), the size of the output image, a measure of distance weighting, and the Region of Interest (ROI) on which to apply the rendering. Looping could be done on the 3D coordinates, or on the 2D reprojection coordinates plus range coordinate. Vectorisation was performed on the 2D reprojection coordinates while the loop on the range coordinates was maintained. Linear 3D interpolation was written out explicitly.

**Listing 12:**
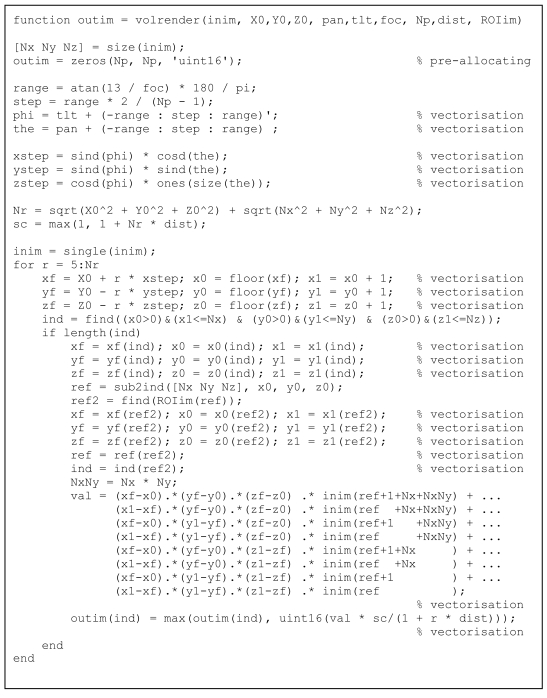
Code for volume rendering.

## RESULTS

**Figure 1:**
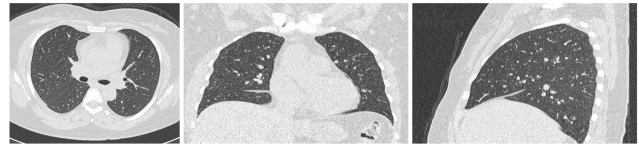
Typical transversal, coronal and sagittal sections of the image used in the testing procedures.

The algorithms were tested on a HP xw4300 Workstation, Pentium 4 at 3.0 GHz with 1 Gb of RAM running Microsoft Windows^®^ XP and MatLab^®^ 7.1. The test image is a Thin Slice CT chest image of a 47-year-old female, 319 slices of 512x512 pixels taken with a Siemens Sensation 16 with pixel spacing of 0.57 mm and a slice thickness of 0.75 mm. Typical cross-sections are shown in Figure 1

The input image was analysed in full resolution and in sub-sampled versions with sampling factors 2 and 4, resulting in image sizes of 512x512x319, 256x256x160 and 128x128x80. Processing time was measured using the MatLab^®^ Profiler for 10 runs of each routine and the average time was listed in Table 1. In a number of cases, the output could not be calculated due to lack of memory; in these cases, obviously no time was recorded.

**Table 1:** Processing time for different algorithms

**Image size:**	128x128x80	256x256x160	512x512x319
**scale1**	1.66	20.19	-.--*
**scale2**	0.53	4.31	34.91
**scale3**	0.02	0.09	0.83
**scale4**	1.19	8.94	74.25
**scale5**	0.11	0.89	7.02
**scale6**	0.08	0.64	5.06
**findlocmax1**	29.44	227.39	1867.70
**findlocmax2**	2.38	-.--*	-.--*
**findlocmax3**	2.31	18.38	-.--*
**imregionalmax**	1.45	12.34	96.20
**bilinterpr2D**	0.03	0.08	0.31
**imresize**	1.48	4.86	19.67
**watershed3D**	6.53	51.09	-.--*
**watershed**	7.83	54.97	-.--*
**volrender**	4.72	33.20	202.25

For the scaling functions, the input range was set to the lung window (-1250 - 250) and the output range to the range of MatLab^®^ for the viewing of floating-point images (0 - 1).

For the functions bilinterp2D and imresize, the 100^th^ slice of the 3D input image was arbitrarily chosen, and the chosen scaling factor was non-integer and significantly larger than two and set to 6.43.

For the watershed, no pre-filtering was applied and the watershed was applied on the original grayscale image.

The volume rendering took pan and tilt angles of 10 degrees, a focal length of 50 pixels and a distance measure of 50. The size of the reprojection was the same as the main size of input image (e.g., 256x256 for the 256x256x160 input image). The location of the camera was slightly outside the volume. The routine was run 10 times with the ROI defined to be the whole volume and 10 times with an ROI centering on the lungs (defined by simple thresholding and morphological filtering), and the average was calculated.

## DISCUSSION

The pre-allocation in the first example (scaling function) produced a performance enhancement of a factor between 3:1 and 11:1, while the vectorisation gave an additional performance enhancement of a factor between 25:1 and 42:1, resulting in a total gain of a factor 83:1 to 201:1. The code gained in readability and not more memory was used. It is clear that code vectorisation offers great advantages when programming in MatLab^®^.

Using conditional statements, the performance enhancement by using vectorisation was only a factor of 2:1 to 7:1, which is still significant. The implicit calculation of indices only slightly speeded up the code (30 to 70%), resulting in a total performance enhancement of a factor of 14:1.

For neighbourhood operations, using an augmented matrix resulted in a gain of a factor 7:1 to 12:1 but at the cost of a prohibitive memory requirement. Even the 256x256x160 image could not be processed. Without the use of an augmented matrix and at the cost of a slight loss in functionality, vectorisation resulted in a gain of a factor 8:1 to 12:1, although the vectorised routine still required more memory than the non-vectorised one. The corresponding built-in function (programmed in C/C++) was faster only by 11 to 60% and with similar memory usage, showing that the MatLab^®^ code can compete with the C/C++ code.

Specialization can have surprisingly good results as shown in the case of 2D bilinear interpolation. A gain by a factor of 15:1 to 60:1 was achieved. In case of building a user interface whereby a large number of interpolations has to be done in a short time, such gain of time might be essential [18].

In the case of watershed interpolation, the implementation in MatLab^®^ ran faster than the implementation in C with 7% to 20% difference in speed but with a number of noteworthy advantages. First, steepest-descent implementation makes it possible to work with floating-point input as in the case of Gaussian blurring, which is essential for multiresolution implementation [19]. Second, the possible return values of rootim and linkim make it possible to quickly and seamlessly implement alternative applications – as preprocessing algorithm, in multiresolution implementation, applied on original gray value image, gradient image or distance transform, and postprocessing like segment merging [20]. But even the simple fact that the MatLab^®^ implementation was roughly as fast as the C implementation shows that MatLab^®^ should not be necessarily regarded as slow and memory-consuming. However, it is essential that good programming practices be applied.

Finally, the volume rendering was slow but similar to an implementation in MEX (not shown here since the MatLab^®^ implementation is the topic of this article) and volume rendering usually *is* slow.

Three particular applications in medical image processing have been discussed: bilinear interpolation (easily expandable to trilinear or bicubic interpolation), watershed segmentation and volume rendering. The code provided can be reused freely in any medical image processing applications and the principles used can serve as examples in the process of improving one’s MatLab^®^ programming techniques.

Although the three methods for MatLab^®^ code optimization i.e., vectorisation, pre-allocation and specialization, were specifically discussed in the context of medical image processing, those techniques are equally applicable in other application domains.

## TO PROBE FURTHER

As mentioned in the introduction, good programming practices when using MatLab^®^ are advocated in the MatLab^®^ user manuals and help files and the relevant references [3-6] can certainly be used to probe further on the issues related to MatLab^®^ vectorisation. In particular, the technical note in [6] gives quite a comprehensive guide to code vectorisation. Bar [3] explains the use of the meshgrid command to help vectorising the processing of 2D and 3D arrays. Eddins [4] explains the use of the find operator to vectorise conditional statements. And the technical note in [5] gives a number of varied valuable suggestions on how to increase the speed of MatLab^®^ code.

## CONCLUSION

MatLab^®^ has usually been tagged a high-level language with a lot of flexibility but inherently slow and memory-consuming, just meant for fast development of algorithms or one-shot applications but not for production environment. The experiments in this article have shown that proper programming techniques should be developed, particularly in the case of medical image processing where data sets (e.g., HRCT data) tend to be big.

Vectorisation and pre-allocation are the most traditional techniques for writing faster MatLab code and are well-documented in the literature and the technical documentation provided by MathWorks. Despite this fact, even some native MatLab functions are written in MEX (C) code while the corresponding MatLab (m) code is just as efficient.

Finally, specialization is an option to consider seriously when specific functions are often used in a very specialised or limited context and execution speed is an issue.
